# Telemedical Approaches to Managing Gestational Diabetes Mellitus During COVID-19: Systematic Review

**DOI:** 10.2196/28630

**Published:** 2021-08-05

**Authors:** Claudia Eberle, Stefanie Stichling

**Affiliations:** 1 Medicine with specialization in Internal Medicine and General Medicine Hochschule Fulda - University of Applied Sciences Fulda Germany

**Keywords:** gestational diabetes, telemedicine, mobile applications, COVID-19, systematic review, digital health, diabetes

## Abstract

**Background:**

In 2019, a new coronavirus emerged in China, and the disease caused by the virus (COVID-19) was rapidly classified as a pandemic. Pregnant women with gestational diabetes mellitus (GDM) are considered to be at risk for severe COVID-19. In the context of the pandemic, there are serious concerns regarding adverse effects on maternal and neonatal outcomes for women with GDM. Effective treatments for patients with GDM are therefore particularly important. Due to contact restrictions and infection risks, digital approaches such as telemedicine are suitable alternatives.

**Objective:**

This systematic review aims to summarize currently available evidence on maternal and offspring outcomes of pregnant women with GDM and COVID-19 and to examine telemedical interventions to improve maternal glycemic control during the COVID-19 pandemic.

**Methods:**

Publications were systematically identified by searching the Cochrane Library, MEDLINE via PubMed, Web of Science Core Collection, Embase, and CINAHL databases for studies published up to March 2021. We sorted the COVID-19 studies by outcome and divided the telemedical intervention studies into web-based and app-based groups. We analyzed case reports (COVID-19) and both randomized and nonrandomized controlled clinical trials (telemedicine). To determine the change in glycated hemoglobin A_1c_ (HbA_1c_), we pooled appropriate studies and calculated the differences in means, with 95% CIs, for the intervention and control groups at the end of the interventions.

**Results:**

Regarding COVID-19 studies, we identified 11 case reports, 3 letters, 1 case series, and 1 retrospective single-center study. In total, 41 patients with GDM and COVID-19 were analyzed. The maternal and neonatal outcomes were extremely heterogeneous. We identified adverse outcomes for mother and child through the interaction of GDM and COVID-19, such as cesarean deliveries and low Apgar scores. Furthermore, we selected 9 telemedicine-related articles: 6 were randomized controlled trials, 2 were clinical controlled trials, and 1 was a quasi-experimental design. In total, we analyzed 480 patients with GDM in the intervention groups and 494 in the control groups. Regarding the quality of the 9 telemedical studies, 4 were rated as strong, 4 as moderate, and 1 as weak. Telemedical interventions can contribute to favorable impacts on HbA_1c_ and fasting blood glucose values in the context of the COVID-19 pandemic. Meta-analysis revealed a mean difference in HbA_1c_ of –0.19% (95% CI 0.34% to 0.03%) for all telemedical interventions, –0.138% (95% CI –0.24% to –0.04%) for the web-based interventions, and –0.305% (96% CI –0.88% to 0.27%) for the app-based interventions.

**Conclusions:**

Telemedicine is an effective approach in the context of COVID-19 and GDM because it enables social distancing and represents optimal care of patients with GDM, especially with regard to glycemic control, which is very important in view of the identified adverse maternal and neonatal outcomes. Further research is needed.

## Introduction

In 2019, a new coronavirus known as SARS-CoV-2 emerged in Wuhan, China; the disease caused by this virus, COVID-19, was classified as a pandemic in a short period of time [1]. Since then, 115,289,961 million people have been diagnosed with COVID-19 worldwide, and 2.564.560 million people had died as of March 5, 2021 [1]. The most vulnerable populations to this virus are those with chronic diseases such as diabetes mellitus (DM) [2-4]. Pregnant women are also considered risk patients [5]. In addition, pregnancy complications such as DM and hypertension have known as a double risk factor of COVID-19 for pregnant women [6]. Pregnant women become particularly vulnerable if they are diagnosed with gestational diabetes mellitus (GDM) and also infected with SARS-CoV-2.

In 2019, in approximately 16% of live births, the mother suffered from hyperglycemia during pregnancy [7]. In total, 84% of those births were associated with GDM [7]. The global prevalence of GDM was between 2.1% and 37.5% as of 2019, depending on screening methods and diagnostic criteria [7]. GDM is diagnosed in the second or third trimester and is defined as not overt diabetes prior to gestation [8]. GDM-complicated pregnancies are associated with adverse maternal and offspring outcomes such as preeclampsia, hypoxia, pregnancy-induced hypertension, type 2 diabetes, obesity and macrosomia, neonatal hypoglycemia, large for gestational age, and adult type 2 diabetes, and cardio-metabolic diseases [9-11]. Intrauterine exposure to increased levels of hyperglycemia “program” the offspring for these lifelong consequences [12-16]. This concept of transgenerational programming (also known as “fetal programming” or “perinatal programming”) leads to perturbation during the development phase by molecular mechanisms, which may lead to dysfunctions in organs and metabolism [12-16].

In the context of the COVID-19 pandemic and with a focus on women with GDM, there are serious concerns regarding adverse effects on maternal, fetal, and neonatal outcomes [17]. Patients with GDM need special care; however, clinical implications of COVID-19 are unexplored [5]. Social distancing and quarantine have ensured that clinic visits are reduced owing to fear of infection [18]. Furthermore, the measures ensure that physical activity is reduced and eating habits change negatively; moreover, there are overall effects on health care and access to medication [18].

Owing to the contact restrictions and restrictions in the health care area, digital solutions are available to ensure close treatment of patients with GDM. National guidelines explicitly recommend telemedicine for the management of GDM during the COVID-19 pandemic in countries such as Canada, Australia, New Zealand, United Kingdom, Germany, Italy, and India [19-22]. The national guidelines advise alternative opportunistic screening strategies with a focus on glycated hemoglobin A_1c_ (HbA_1c_), random plasma glucose (RPG), and fasting blood glucose (FBG).

Diabetes technology includes hardware, software, and technical devices that help to control the disease [23]. Telemedical treatments show great potential in clinical diabetes management [24-26]. Telemedicine characterizes the use of communication technologies to improve patient outcomes by increasing access to care and medical information [23]. Telemedicine overcomes geographical and physical limits, improves health care access, and enhances health-related outcomes [23].

More clinical and scientific analyses are urgently needed in the context of COVID-19, GDM, and digital therapy options. It is generally advisable to draw up a general pandemic guideline with corresponding recommendations for action, which can also be useful in case of other pandemics.

Against this background, we examine pregnant women with GDM in more detail in the context of the COVID-19 pandemic and with a view to telemedical treatment options. In this new research area, it is important to analyze previous literature and provide suggestions for further research. Due to the limited evidence in this field, this systematic review aimed to summarize currently available evidence on maternal and offspring outcomes of pregnant women with GDM and COVID-19 and to examine telemedical treatment methods to improve maternal glycemic control during the COVID-19 pandemic. With this analysis, we would like to set a first milestone and a starting point for further research in the areas of gestational diabetes, COVID-19, and telemedicine.

## Methods

### Search Strategy

In general, when creating the systematic review and analyzing the studies, we followed the *Cochrane Handbook for Systematic Reviews of Interventions* [27].

Publications were systematically identified by searching the Cochrane Library, MEDLINE via PubMed, Web of Science Core Collection, Embase, and CINAHL databases for studies published up to March 2021.

We conducted two systematic searches to address our respective research questions. One focused on the topic of GDM and COVID-19, and the other focused on the topic of GDM and telemedicine. The search strategies are shown in Multimedia Appendix 1. After searching the databases, we removed duplicate entries, screened titles and abstracts for suitability, and then read the full texts. The studies were selected by two independent reviewers.

#### COVID-19

This search was conducted using the following keywords: (“gestational diabetes mellitus”) AND (“COVID-19” OR “coronavirus” OR “SARS-CoV-2”). Medical Subject Headings (MeSH) and Embase Subject Headings terms as well as title/abstract terms were searched. In addition, we manually searched reference lists.

#### Telemedicine

This search was conducted using the following keywords: (“gestational diabetes mellitus”) AND (“telemedicine” OR “telemonitoring” OR “telemetry” OR “mHealth” OR “mobile applications” OR “smartphone”). MeSH and Embase Subject Headings terms as well as title/abstract terms were searched. In addition, we manually searched reference lists.

### Inclusion Criteria

#### COVID-19

Studies that met the following inclusion criteria were selected: peer-reviewed; published in English or German; observational, cohort, and clinical studies, case reports/series, letters, and comments reporting maternal, fetal, and neonatal outcomes in pregnant women with GDM and COVID-19.

Because COVID-19 emerged at the end of 2019 and is still quite unexplored, especially for special target groups such as patients with GDM, few studies are available to date. We therefore considered various study designs (including case reports and letters) and included all papers that reported on patients with COVID-19 and GDM, even if GDM was not explicitly the focus of the respective paper. Furthermore, we did not impose any restrictions on maternal, fetal, or neonatal outcomes.

#### Telemedicine

Articles that met the following inclusion criteria were selected: peer-reviewed; published in English or German; clinical controlled trials (CCTs) and randomized controlled trials (RCTs) examining telemedical treatment for pregnant women with GDM and reporting on the maternal outcomes of HbA_1c_, FBG, or RBG.

Telemetry was defined as remote recording and transmission of patient data via a telecommunications system to a health care provider to provide clinical support and improve health outcomes [23]. We included video calls, telephone calls, internet/web-based platforms, and smartphone/mobile app–based interventions.

### Exclusion Criteria

#### COVID-19

The following articles were excluded: papers that did not specify the type of diabetes, and articles that did not report maternal or offspring outcomes and focused instead on GDM screening/management/diagnosis, anxiety and stress during lockdown, or prevention of GDM.

#### Telemedicine

The following articles were excluded: posters, comments, letters, study protocols, and proceedings; studies that did not specify the type of diabetes; studies that described technologies only; studies that pooled data with other technologies and other diseases; and papers focusing on GDM prevention or GDM diagnosis.

### Data Extraction

We extracted the year of publication, study region, study design, patient characteristics, intervention details for the telemedical studies (type of technology, intervention and control group, sample size), outcomes, and main results.

### Data Synthesis and Analysis

#### COVID-19

We sorted the COVID-19 studies by outcome. We divided the outcomes into maternal and offspring outcomes. Where possible, we calculated the means (Apgar score and birth weight).

#### Telemedicine

We divided the telemedical studies into two groups: web-based and app-based. Web-based interventions are based on websites and the internet, with asynchronous communication between patients and health care providers. App-based interventions are based on smartphone and mobile phone apps.

We conducted a meta-analysis to determine the impact of the interventions on the HbA_1c_ concentration because this was the most frequently studied outcome. We used Excel (Microsoft Corporation) for pooling the data and for calculations, and we took into account all studies that provided complete information. In the calculations, we considered web-based and app-based studies. For determining the change in HbA_1c_ (%), we pooled appropriate studies and calculated the differences in the means, with 95% confidence intervals, for the intervention and control groups at the end of the interventions. Based on the HbA_1c_ mean differences and confidence intervals, we created a forest plot.

### Assessment of Risk of Bias

A quality appraisal of the studies was performed to determine the risk of bias. We used the valid and reliable Effective Public Health Practice Project (EPHPP) tool [28] for the appraisal of randomized and nonrandomized clinical trials on health-related topics. EPHPP consists of the following components: selection bias, study design, confounders, blinding, data collection methods, and withdrawals and dropouts. The tool classifies the study quality ranging between strong, moderate, and weak. The assessment was performed by one reviewer.

## Results

### COVID-19

#### Search Results and Trial Flow

The literature search yielded 228 citations, of which 136 unique citations were screened based on title and abstract (Multimedia Appendix 2). Then, we screened 19 articles based on the full texts. We added 1 study (Blauvelt et al [29]) through manual research. Finally, we selected 16 appropriate COVID-19–related articles in this systematic review [3,5,29-42]. Of these, 11 were case reports, 3 were letters, 1 was a case series, and 1 was a retrospective single-center study. In total, 41 patients with GDM were analyzed. An overview of all included studies is provided in Multimedia Appendix 3.

Overall, the reported outcomes are very heterogeneous, and there are only a few overlaps of the same outcomes in the studies. It should also be noted that the papers are not very detailed and generally contain little information, which is related to the fact that at the beginning of the COVID-19 pandemic, papers can be published more quickly and in a simplified form.

#### Maternal Outcomes

The most common COVID-19 symptoms were fever (n=12), cough (n=13), dyspnea (n=4), shortness of breath (n=3), diarrhea (n=2), and vomiting (n=2).

In total, 22 women required cesarean deliveries, including 3 emergency cesarean deliveries. Furthermore, 5 women were admitted to the intensive care unit (ICU). Blauvelt et al [29] reported lung overdistension, hyperglycemia after antenatal corticosteroid administration, and mechanical ventilation (n=1 woman). Cooke et al [30] noticed that 1 patient experienced psychiatric sequelae postoperatively. In addition, Vlachodimitropoul et al [31] outlined a postpartum hemorrhage of 1.5 L, controlled with uterine artery ligation and B-Lynch compression alongside uterotonics and blood products (n=1 patient). Govind et al [32] reported that, following delivery, a patient continued to desaturate (80%-85%) on 100% oxygen.

Kleinwechter and Laubner [3] noted vaginal bleeding and that the patient was fever-free at day 6, with oxygen requirement declining (n=1 woman).

Moreover, Oliva et al [33] outlined that the status of the patient (fever, dyspnea, respiratory status worsening from oxygen saturation as measured by pulse oximetry [SpO_2_] 95%-92%) improved rapidly post–cesarean delivery (2 hours after delivery SpO_2_ to the low 90th percentile on room air, which improved to 100% on 15 L/min of oxygen) and that the cesarean delivery was uncomplicated. Uzel and Lakhno [35] found leukocytosis (19 × 103/μL) and mild anemia (hemoglobin 10.1 g/dL) in the clinical blood test (n=1 woman). Activated partial thromboplastin time, prothrombin time, and international normalized ratio were prolonged, levels of C-reactive protein (179.7 mg/L) and procalcitonin (0.15 ng/ml) were raised, and low oxygen saturation (75%) was detected. The authors reported that the patient died (35 year-old woman with obesity, lymphopenia, hyperglycemia, and trace proteinuria). D’Ambrosi et al [37] outlined that 6 women (in a retrospective single-center study) did not require ICU admission or mechanical ventilation, but 4 women underwent elective cesarean deliveries.

In addition, Fontanella et al [38] reported that there were no adverse maternal outcomes and the women left the hospital after 5 days because of rapid improvement. Kleinwechter et al [5] (n=21 women) showed 2 admissions to the ICU, and in 4 cases, the women received symptomatic COVID-19–associated therapy. No invasive ventilation was required, and 56% of the cases were cesarean deliveries (9/16).

#### Offspring Outcomes

The mean Apgar score (1 minute) was 5.9 in 8 newborns [29-33,35,36,39]. In addition, the mean Apgar score (5 minutes) was 7.5 in 17 newborns (including 1 set of triplets) [29-33,35-37,39]. The birth weight [30,32-37,40,41] was between 1250 g [40] (a triplet) and 4165 g [32], whereas the mean birth weight was approximately 2621.29 g (n=17 newborns).

Blauvelt et al [29] reported that the newborn evolved respiratory distress syndrome and laboratory test results showed leukopenia, neutropenia, lymphopenia, mild acidosis, and normal lactate. A SARS-CoV-2 test was negative, and at 16 days of life, the neonate was clinically stable on high-flow nasal cannula at 3 L/min and 21% fraction of inspired oxygen (FiO_2_). Govind et al [32] observed viral pneumonia on day 6, but the newborn recovered well and was ventilated for 10 days.

Moreover, Oliva et al [33], Uzel and Lakhno [35], Majachani et al [39] and Rabiei et al [40] noted admissions to the neonatal intensive care unit (NICU) (n=6 newborns). In total, 2 newborns needed supplement oxygen [32,33]. Additionally, 7 children tested negative for COVID-19 after their births [29,35,36,40,41], and 1 had a positive nasopharyngeal swab after 24 hours. Pulinx et al [42] reported the death of 2 fetuses (twins). Cooke et al [30] and Fontanella et al [38] observed no adverse neonatal outcomes.

### Telemedicine

#### Search Results and Trial Flow

The literature search yielded 408 citations, of which 348 unique citations were screened based on title and abstract (Multimedia Appendix 3). Then, we screened 14 articles based on the full texts. Finally, we selected 9 appropriate telemedicine-related articles [43-51] in this systematic review. Of these, 6 were RCTs, 2 were CCTs, and 1 was a quasi-experimental design.

In total, we analyzed 480 patients in the intervention groups and 494 in the control groups. The interventions were grouped into web-based and app-based interventions. We did not find any video consultations, and we did not find studies that reported on the outcome of RPG. In total, we analyzed 6 web-based and 3 app-based telemedicine interventions.

### Quality Assessment

Furthermore, 4 studies were rated as strong, 4 were rated as moderate and 1 as weak quality studies. The quality appraisals are presented in Multimedia Appendix 4.

Table 1 provides an overview of the characteristics and outcomes of the telemedicine studies. Multimedia Appendix 5 contains a PRISMA (Preferred Reporting Items for Systematic Reviews and Meta-Analyses) checklist for the systematic review.

**Table 1:** Characteristics and outcomes of telemedicine studies.

Author (year), location	Design	Patients, n	Type of technology	Intervention details	Outcomes	*P* value
Homko et al (2007), United States [44]	RCT^a^	IG^b^: 34 CG^c^: 29	Web-/internet-based	Web-based management interactive system: data transmission at least 3 times/week, messaging between health care professionals and patients Control: usual care + paper logbooks	HbA_1c_^d^ (change) (%) IG: 6.1 (0.8) CG: 6.2 (2.2) FBG^e^ (change) (mg/dL) IG: 90.8 (11.8) CG: 88.6 (9.5)	Not reported
Homko et al (2012), United States [43]	RCT	IG: 40 CG: 40	Web-/internet-based	Web-based management interactive system: interactive voice response telephone communication, auto-reminders Control: logbook reviewed at clinic visits Both: clinic visits every 2 weeks and weekly from 36 weeks’ gestation	FBG (change) (mg/dL) IG: 91.5 (10.5) CG: 94.3 (0.26)	.26
Kim et al (2019), Korea [46]	Quasi-experimental study	IG: 22 CG: 22	Web-/internet-based	Web-based system: weekly web-based health diary, transmitting daily FBG, individual education Control: received nutrition education	HbA_1c_ (change) (%) IG: 5.0 (0.2) CG: 5.3 (0.2) FBG (change) (mg/dL) IG: 78.8 (8.4) CG: 80.9 (8.4)	Both not significant (not reported)
Given et al (2015), United Kingdom [45]	RCT	IG: 24 CG: 26	Web-/internet-based	Usual care and telemedicine hub with website, mobile phone and landline, weekly feedback Control: face-to-face clinic visits every 2 weeks, self-monitoring of blood glucose	HbA_1c_ IG: 34.04 (3.23) mmol/mol (5.26% increase) CG: 33.84 (2.88) mmol/mol (5.25% increase)	Not reported
Dalfra et al (2009), Italy [47]	CCT^f^	IG: 88 CG: 115	Web-/internet-based	Transmission of glycemic data through telephone receiver and feedback, monthly clinic visits Control: not adequately described	HbA_1c_ (change) (%) IG: 5.1 (0.6) CG: 5.3 (0.5)	.008
Perez-Ferre et al (2010), Spain [48]	RCT	IG: 50 CG: 50	Web-/internet-based	Internet and app–based via SMS text messaging: transmission of self-monitoring data, SMS text message communication between patients and health care professionals Control: face-to-face outpatient clinic visits	HbA_1c_ (change) (%) IG: 5.3 (0.4) CG: 5.4 (0.4)	Not reported
Mackillop et al (2014), United Kingdom [50]	RCT	IG: 101 CG: 102	App-based	Mobile phone-based blood glucose management solution: health app (data transmission and feedback at least 3 times per week), clinic visits every 4 to 8 weeks	HbA_1c_ 0.02% rise per 28 days in the intervention group and 0.03% rise per 28 days in the control group No statistically significant difference (intervention vs control: –0.01%, 95% CI –0.05 to 0.03)	Not reported
Guo et al (2019), China [49]	RCT	IG: 64 CG: 60	App-based	Usual care + app “Dnurse” for data transmission and feedback, and educational information about gestational diabetes mellitus Control: standard outpatient treatment	HbA_1c_ (change) (%) IG: 4.7 (0.2) CG: 5.3 (0.3) FBG (change) (mg/dL) IG: 4.9 (0.9) CG: 5.0 (0.8)	HbA_1c_: .001 FBG: .74
Yang et al (2018), China [51]	CCT	IG: 57 CG: 50	Smartphone/WeChat	Smartphone-based telemedicine system (WeChat app) and articles providing continuous health education, cloud database Control: Usual care and health education	FBG (change) (mg/dL) IG: 4.31 (0.75) CG: 5.31 (1.29)	<.001

^a^RCT: randomized controlled trial.

^b^IG: intervention group.

^c^CG: control group.

^d^HbA_1c_: glycated hemoglobin A_1c_.

^e^FBG: fasting blood glucose.

^f^CCT: controlled clinical trial.

#### Effects on HbA_1c_ Values

The effects of the interventions based on the comparison of HbA_1c_ levels between the intervention and control groups at the end of the study were analyzed. Dalfra et al [47] (strong quality) and Guo et al [49] (strong quality) indicated clear and significant improvements of HbA_1c_ values (*P*<.001 [49] and *P*=.008 [47]). Our findings are based on 5 web-based [44-48] and 2 app-based [49,50] studies. The meta-analysis revealed a mean effect size of –0.138% (95% CI –0.24 to –0.04) for web-based interventions and –0.305% (95% CI –0.88 to 0.27) for app-based interventions.

In general, the meta-analysis demonstrated a mean difference in HbA_1c_ of –0.19% (95% CI 0.34 to 0.03) for all telemedical interventions (n=7). Multimedia Appendix 6 provides the forest plot and the calculations and data for the meta-analysis.

#### Effects on FBG Values

In total, 2 of 3 studies reported improvements in FBG levels by web-based interventions. Homko et al [43] (moderate quality) outlined enhancements in their intervention group compared to the control group (intervention, 91.5 [10.5] mg/dL, vs control, 94.3 [0.26] mg/dL, *P*=.26). In addition, Kim et al [46] (weak quality) showed lower FBGs in the intervention group (78.8 [8.4] mg/dL) compared to the control group (80.9 [8.4] mg/dL), but the difference was not significant between groups (*P* value not reported).

Furthermore, all app-based studies (n=2) reported clear decreases in FBG values by implementing app-based telemedicine interventions. Yang et al [51] (moderate quality) indicated that a smartphone-based telemedicine system (WeChat app) was significantly associated with an obvious improvement in FBG (intervention 4.31% (0.75) versus control 5.31% (1.29), *P*<.001). Moreover, Guo et al [49] (strong quality) showed improvements compared to the controls (intervention 4.9% (0.9) vs control 5.0% (0.8), *P*=.74).

## Discussion

### Principal Results

This systematic review indicated that telemedicine interventions, especially web-based and app-based treatments, can contribute to favorable impacts on HbA_1c_ and FBG values in the context of the COVID-19 pandemic. Meta-analysis revealed a mean difference in HbA_1c_ of –0.19% (95% CI 0.34 to 0.03) for all telemedical interventions (n=7), –0.138% (95% CI –0.24 to –0.04) for web-based interventions (n=5), and –0.305% (96% CI –0.88 to 0.27) for app-based interventions (n=2).

Digital treatment approaches are an innovative alternative to conventional GDM therapies, particularly due to social distancing, quarantine, and the risk of infection for pregnant women. Interest in telemedicine is increasing because of benefits such as the lack of need for physical presence as well as cost and time benefits [52,53].

Optimal glycemic control through telemedicine can mitigate negative consequences for mother and child. Patients with GDM and COVID-19 need special care. Using the current evidence, we were able to identify and analyze adverse outcomes for mother and child through the interaction of GDM and COVID-19, such as cesarean deliveries, admission to the (neonatal) ICU, ventilation, and low Apgar scores. The long-term consequences for mother and child are currently unknown. Unfortunately, the papers available are often short, are not detailed, and only consist of case reports and letters. Clinical studies with strong methodologies on various outcomes in the context of COVID-19 and GDM are urgently needed to determine the clinical implications. Furthermore, the outcomes were extremely heterogeneous, and quantitative analysis and synthesis were therefore difficult.

As mentioned, early therapeutic strategies are required to improve the management of GDM effectively, because GDM can contribute to long-term consequences for the child by transgenerational programming. Transgenerational programming is a perturbation during development phases that can lead to “programming errors” in organ functions and metabolic regulation. This leads to diseases in later life, such as non–insulin-dependent diabetes, obesity, hypertension, and cardiovascular disease [54]. GDM can contribute to these programming errors and to long-term consequences for the child [54]. GDM occurs over a limited period of time; therefore, little time is available to understand and treat the condition, and effective therapies targeting glycemic and metabolic control are thus necessary [55].

Overall, telemedical approaches clearly improve the glycemic control of women with GDM and thus lead to a positive effect regarding transgenerational programming. Telemedicine effectively enhances the management of GDM in the context of the COVID-19 pandemic. Other reviews by us [24-26] showed that telemedical therapy can improve glycemic control, decrease the number of scheduled and unscheduled hospital visits, and improve several fetal and neonatal short-term outcomes.

With regard to different types of telemedical interventions, we were able to identify web-based and app-based interventions; however, fewer interventions were app-based despite the good operability of smartphone apps. We could not find other technologies, such as videoconferences, in the GDM context, but these technologies show great potential for other types of diabetes.

### Limitations

The national guidelines as well as different threshold values for the diagnosis of GDM affect the findings and must be considered. With different definitions of GDM, participants may not be precisely comparable. A uniform global definition of GDM would be necessary to address this issue.

Moreover, because COVID-19 is a rapidly developing topic, the COVID-19 studies are methodologically weak, are not very detailed, and were published quickly to rapidly publish important findings and generate evidence in the context of the new COVID-19 pandemic. This limitation must be taken into account regarding this analysis.

In addition, the maternal and offspring outcomes were extremely heterogeneous. More studies are needed that bring patients with GDM into focus. Larger sample sizes are necessary. In further studies, it is advisable to focus on maternal, fetal, and neonatal outcomes, which are also considered in other GDM studies (when analyzing therapy options for patients with GDM) and are particularly relevant to health, such as cesarean deliveries, admission to the ICU, glycemic control (hyperglycemia, fasting blood glucose, HbA_1c_, etc), fetal distress, Apgar score, birth weight, and respiratory distress syndrome. With regard to COVID-19–relevant outcomes, our analysis has shown that in particular, COVID-19 symptoms, ventilation, COVID-19 therapy, SARS-CoV-2 testing of newborns, nasopharyngeal swabs, and supplemental oxygen are relevant and need further investigation.

### Comparison With Prior Work

To our knowledge, we are the first group to perform a systematic review on GDM, COVID-19, and telemedicine. Other reviews and meta-analyses on GDM and telemedicine reported similar positive results regarding telemedicine care in gestational diabetes management [56,57]. Xie et al [58] showed that, compared to usual care, telemedicine interventions can decrease the glycemic levels of patients with GDM effectively and reduce the risk complications (HbA_1c_, mean difference −0.70, *P*<.01, and FBG, mean difference –0.52, *P*<.01). However, evidence of clinical effectiveness of telemetric interventions on GDM management is still lacking, particularly regarding app-based and video-based interventions.

### Conclusions

This review sets a first milestone and a starting point for further research in managing patients with GDM during the COVID-19 pandemic.

Pregnant women with GDM are at increased risk of a severe course of COVID-19. In the context of the COVID-19 pandemic, a shift to digital therapy approaches, such as telemedicine, is taking place in medical practices. At this point in time, analyses and conclusions on adverse outcomes in women with COVID-19 and GDM are scarce; however, we have summarized the first evidence available. Further research is needed to understand the epidemiology and health care interventions needed to most effectively treat women with COVID-19 and GDM. In addition, the transgenerational interplay between COVID-19 and GDM needs further investigation, and evidence-based recommendations are needed.

Telemedicine is a modern and effective approach in the context of COVID-19 and GDM, as it reduces contact and enables patients with GDM to still be optimally treated with regard to adverse pregnancy outcomes. Other telemetric approaches, such as video consultations, need to be investigated more closely, and intervention effects in relation to RPG should also be examined.

## Appendix

Multimedia Appendix 1 Search strategies.

Multimedia Appendix 2 PRISMA (Preferred Reporting Items for Systematic Reviews and Meta-Analyses) flow charts.

Multimedia Appendix 3 Overview of COVID-19 studies.

Multimedia Appendix 4 Quality assessments.

Multimedia Appendix 5 PRISMA (Preferred Reporting Items for Systematic Reviews and Meta-Analyses) checklist.

Multimedia Appendix 6 Forest plot.

## Abbreviations

CCT: clinical controlled trial
DM: diabetes mellitus
EPHPP: Effective Public Health Practice Project
FBG: fasting blood glucose
FiO2: fraction of inspired oxygen
GDM: gestational diabetes mellitus
HbA_1c_: glycated hemoglobin A_1c_
ICU: intensive care unit
MeSH: Medical Subject Headings
NICU: neonatal intensive care unit
PRISMA: Preferred Reporting Items for Systematic Reviews and Meta-Analyses
RCT: randomized controlled trial
RPG: random plasma glucose
SpO_2_: oxygen saturation as measured by pulse oximetry
