# Supplementary material for: Telemedical Approaches to Managing Gestational Diabetes Mellitus During COVID-19: Systematic Review
Source: JMIR Pediatr Parent. 2021 Aug 5;4(3):e28630. doi: 10.2196/28630 (PMC8345174; doi:10.2196/28630)
Supplement: Multimedia Appendix 1 [file pediatrics_v4i3e28630_app1.docx]

**Multimedia Appendix 1. Search strategies.**

|  | PUBMED | EMBASE | COCHRANE | CINAHL | WEB OF SCIENCE  CORE COLLECTION |
| --- | --- | --- | --- | --- | --- |
| **KEYWORDS** | gestational diabetes, telemetry, telemedicine, telemonitoring | pregnancy diabetes mellitus, gestatioanl diabetes, telemetry, telemedicine, telemonitoring | gestational diabetes, telemetry, telemedicine, telemonitoring | gestational diabetes, telemetry, telemedicine, telemonitoring | gestational diabetes, telemetry, telemedicine, telemonitoring |
| **FILTERS** | Published until 2021/03/01,  English; German | Published until 2021/03/01,  English; German | Published until 2021/03/01,  English; German | Published until 2021/03/01,  English; German | Published until 2021/03/01,  English; German |
| **STRATEGY** | (("gestational diabetes"[Title/Abstract]) OR diabetes, gestational[MeSH Terms]) AND ("Telemetry"[Mesh]) OR "Telemedicine"[Mesh]) OR telemonitoring[Title/Abstract]) OR “mHealth”[Title/Abstract]) OR “mobile application”[Mesh]) OR “smartphone”[Mesh])) | ((‘pregnancy diabetes mellitus’/exp OR ‘gestational diabetes’: ab,ti) AND (‘telemetry’/exp OR ‘telemedicine’/exp OR ‘telemonitoring/exp OR ‘mHealth’:ab,ti OR ‘mobile application’/exp OR ‘smartphone’/exp)) | ((MeSH [Diabetes, Gestational] OR “gestational diabetes”:ti,ab) AND (MeSH [Telemedicine] OR MeSH [Telemetry] OR “telemonitoring”:ti,ab OR MesH [mobile application] OR MesH [smartphone] OR “mHealth”:ti,ab)) | ((TI “gestational diabetes” OR AB “gestational diabetes”) AND (MH telemedicine OR MH telemetry OR TI telemoritoring OR AB telemonitoring OR TI mHealth OR AB mHealth OR MH smartphone OR MH mobile application)) | ((TOPIC “gestational diabetes”) AND (TOPIC telemetry OR TOPIC telemedicine OR TOPIC telemonitoring OR TOPIC mHealth OR TOPIC mobile application OR TOPIC smartphone)) |

# **Search strategy COVID-19.**

|  | PUBMED | EMBASE | COCHRANE | CINAHL | WEB OF SCIENCE  CORE COLLECTION |
| --- | --- | --- | --- | --- | --- |
| **KEYWORDS** | gestational diabetes, COVID-19, SARS-CoV-2 | coronavirus disease 2019, severe acute respiratory syndrome coronavirus, pregnancy diabetes mellitus | COVID-19, SARS-CoV-2, coronavirus, gestational diabetes | COVID-19, SARS-CoV-2, gestational diabetes | COVID-19, SARS-CoV-2, gestational diabetes mellitus |
| **FILTERS** | Published until 2021/03/01,  English; German | Published until 2021/03/01,  English; German | Published until 2021/03/01,  English; German | Published until 2021/03/01,  English; German | Published until 2021/03/01,  English; German |
| **STRATEGY** | (diabetes, gestational[MeSH Terms])) AND (("SARS-COV-2"[Title/Abstract]) OR ("COVID-19" [Supplementary Concept])) | ((‘pregnancy diabetes mellitus’) AND (‘coronavirus disease 2019’/exp OR 'severe acute respiratory syndrome coronavirus 2'/exp)) | ((MeSH [Diabetes, Gestational]) AND (“COVID-19”:ti,ab OR “SARS-CoV-2”:ti,ab OR “coronavirus”:ti,ab)) | ((TI “gestational diabetes” OR AB “gestational diabetes”) AND (TI “SARS-CoV-2” OR AB “SARS-CoV-2” OR MH “COVID-19)) | (((TOPIC “diabetes mellitus” AND “pregnancy) OR (TOPIC “gestational diabetes mellitus”)) AND ((TOPIC “COVID-19” OR TOPIC “SARS-CoV-2”))) |
