# Supplementary material for: Telemedical Approaches to Managing Gestational Diabetes Mellitus During COVID-19: Systematic Review
Source: JMIR Pediatr Parent. 2021 Aug 5;4(3):e28630. doi: 10.2196/28630 (PMC8345174; doi:10.2196/28630)
Supplement: Multimedia Appendix 2 [file pediatrics_v4i3e28630_app2.docx]

**Multimedia Appendix 2.** PRISMA (Preferred Reporting Items for Systematic Reviews and Meta-Analyses) flow charts.

**PRISMA flow chart COVID-19 studies (adapted from Moher et al. 2009).**

Citations identified through database searching

(n=228)

n=53 (MEDLINE via PubMed)

n=116 (EMBASE)

n=12 (CINAHL)

n=0 (Cochrane Library)

n=47 (Web of Science Core Collection)

March 2021

Citations excluded based on
title/abstract (n=117)

Other topics (GDM screening/ management/ diagnosis, anxiety, stress, not COVID-19-related, prevention of GDM etc.) (n=71)

Wrong design (poster, study protocols, proceedings papers etc.) (n=23)
Wrong population (not GDM patients) (n=23)

## Identification

## Screening

Citations screened without duplicates
(n=136)

Publications excluded (n=4)

No access (n=1)

Wrong population (not GDM patients, only pregnant women) (n=3)

Studies with full-text assessed for eligibility
(n=19)

## Eligibility

After assessing
full-text studies
(n=15)

Manual research
(n=1)

Finally included in
this review
(n=16)

## Included

GDM: Gestational diabetes mellitus.

**PRISMA flow chart telemedicine studies (adapted from Moher et al. 2009).**

Citations identified through database searching

(n=408)

n=58 (MEDLINE via PubMed)

n=221 (EMBASE)

n=26 (CINAHL)

n=14 (Cochrane Library)

n=89 (Web of Science Core Collection)

March 2021

## Identification

Citations excluded based on
title/abstract (n=334)

Other topics (not telemedicine-related, prevention/diagnosis of GDM, only presentation of the technology etc.) (n=163)

Wrong design (poster, study protocols, proceedings papers etc.) (n=119)
Wrong population (not GDM patients) (n=52)

## Screening

Citations screened without duplicates
(n=348)

Publications excluded (n=5)

No access (n=1)

Wrong population (n=4)

## Eligibility

Studies with full-text assessed for eligibility
(n=14)

Finally included in
this review
(n=9)

## Included

GDM: Gestational diabetes mellitus.
