# Supplementary material for: Telemedical Approaches to Managing Gestational Diabetes Mellitus During COVID-19: Systematic Review
Source: JMIR Pediatr Parent. 2021 Aug 5;4(3):e28630. doi: 10.2196/28630 (PMC8345174; doi:10.2196/28630)
Supplement: Multimedia Appendix 3 [file pediatrics_v4i3e28630_app3.pdf]

Overview of COVID-19 and GDM studies.

Author, Year	Location	Study design	Pregnant women, n	Patient characteristics and medical history	COVID-19	Maternal outcomes	Offspring outcomes
**Blauvelt et al 2020**	USA	Case report	1	• 34- year-old pregnant women with coronavirus infection, 28 weeks of gestation<br>• Asthma, A2 gestational diabetes, obesity, three prior cesarean delivery<br>• Vital signs at admission: temperature 36.9°C, blood pressure 117/66 mm Hg, heart rate 110 beats per minute, respiratory rate 30–40/min, and SpO_2 93% on non-rebreather mask at 15 L/min, transitioned to high-flow nasal cannula, Initial PaO_2/FiO_2 ratio was 163, plateau pressure 15 cmH_2O, static compliance 80 mL/cmH_2O and calculated dead space fraction 42%	• Fever, malaise, myalgias, cough, shortness of breath<br>• ongoing hypoxemia (pH 7.41, $PaCO_2$ 26 mm Hg, PaO_2 64 mm Hg, HCO_3 16 mmol/L).<br>• Higher risk for mortality and morbidity in pregnant women and Covid-19<br>• Acute respiratory distress syndrome<br>• ICU admission, intubation<br>• Placenta showed acute chorioamnionitis<br>• Negative IgM and IgG<br>• incremental titration of positive end-expiratory pressure from 10 to 16 cmH_2O<br>• FiO_2 requirement remained high at 80% and her static compliance worsened	• lung overdistension, evidenced by decreased lung compliance and worsened hypercarbia indicating increased dead space<br>• develop hyperglycemia after antenatal corticosteroid administration<br>• required prolonged mechanical ventilation (until postpartum day 10)<br>• Shortly before delivery: FiO_2 increased to 100%, inhaled nitric oxide initiated at 20 ppm to temporarily improve oxygenation perioperatively, PaO_2 improved to 159 mm Hg (from 94 mm Hg).<br>• Following days PaO_2/FiO_2 ratio improved, lung mechanics remained relatively stable<br>• Extubated on postoperative day 10	• Intubated because of Respiratory distress syndrome<br>• Diffuse bilateral granular/ hazy opacities concerning for mild edema/ envolving respiratory distress syndrome<br>• Laboratory test results showed leukopenia, neutropenia, lymphopenia, mild acidosis, and normal lactate<br>• Negative SARS-CoV-2 test and IgG and IgM<br>• 1920g<br>• Apgar score 4->8<br>• Clear amniotic fluid<br>• At 16 days of life, neonate was clinically stable on high-flow nasal cannula at 3 L/min and 21% FiO_2<br>• normal findings on neurologic examination.
**Cooke et al 2020**	UK	Case Report	1	• 28-year-old,<br>• 28^{+6} weeks of gestation, GDM, Asian	• fever, cough, diarrhoea and vomiting, was tachypnoeic, and bibasal pulmonary infiltrates supported a presumed SARS-CoV-2 diagnosismechanical ventilation because tachypnoeic, c-section	• recovered rapidly<br>• experienced psychiatric sequelae post-operatively	• no complication or bad outcome mentioned, birth weight 1400g, Apgar scores 1 -> 3
**Vlachodimitropoul et al 2020**	Canada	Case Report	1	• 40-year-old women<br>• Gravida 2 para 1<br>• Familia neutropenia diagnosed in infancy (by admission neutropenia (0.1-0.3 × 10^9/L))<br>• GDM<br>• lymphocyte count nadirs of 0.16	• admitted at 35 + 3 weeks' gestation with cough and pyrexia<br>• Normotensive, tachycardic (110-121 beats/min), febrile (39°C), with normal oxygen saturation in room air<br>• SARS-CoV-2 was confirmed by polymerase chain reaction (PCR) on a nasopharyngeal swab<br>• Normal chest X-ray<br>• Over 48 hours, progressive thrombocytopenia, declining fibrinogen, rising activated partial thromboplastin time with concomitant improvement	• sustained a postpartum hemorrhage of 1.5 L controlled with uterine artery ligation and B-Lynch compression, alongside uterotonics and blood products (tranexamic acid 2 g, fibrinogen 4 g, cryoprecipitate 10 units)<br>• C-section was performed	• obstetrical ultrasound demonstrated a well-grown fetus<br>• fetal heart rate monitoring was unremarkable<br>• healthy male infant was delivered, weighing 2.93 kg (Apgars of 9 and 9 at 1 and 5 minutes)

Author, Year	Location	Study design	Pregnant women, n	Patient characteristics and medical history	COVID-19	Maternal outcomes	Offspring outcomes
					in neutrophil count, responding to G-CSF<br>• Significantly elevated D-dimers were observed<br>• Description of mild transaminitis (underlaying infection)<br>• Coagulopathy improvement was noted on postoperative day		
**Govind et al 2020**	UK	Letter	1	• 36-years-old, 38 w.o.g, Mode of delivery: Em C-Section, diet controlled GDM	• Pneumonia, respiratory distress, Chest Xray revealed right basal consolidation and lymphopenia was noted (0.92 × 109/l; normal: 1.2–3.6)	• Following delivery, patient continued to desaturate (80–85 %) on 100 % of oxygen<br>• transferred to a tertiary centre for extracorporeal membrane oxygenation (ECMO)	• Birth weight: 4165 g<br>• Viral pneumonia day 6 but recovered well<br>• Apgar scores 5-> 9<br>• Ventilated for 10 days<br>• Developed pyrexia and exhibited signs of pneumonia on 6th day
**Kleinwechter / Laubner 2020**	Germany	Case Report	1	• 28-year-old pregnant women (Gravida 4, Para 2)<br>• GDM diagnosed in 25th w.o.g 75g-oGTT: 97 mg/dl fasted, 189 mg/dl after 1 h, 197 mg/dl after 2 (5.4 / 10.5 / 9.9 mmol/l)<br>• Admitted to University Hospital Freiburg (9nternistically)<br>• Day before admission, RT-PCR throat swab was positive.	• >39 °C fever, cough, loss of appetite<br>• Thrombocytopenia and lymphocytosis, elevated levels of LDH, creatine kinase, CRP and IL-6<br>• Three days after admission increasing dyspnoea with oxygen demand up to 5 l/min (by mask)<br>•	• Deep-seated placenta occurred vaginal bleeding (temporary transfer to the delivery room and monitoring under tocolysis)<br>• Fever-free at day 6, oxygen requirement declining<br>• First control pharyngeal smear negative	
**Oliva et al 2020**	USA	Case Report	1	• 35-year-old pregnant women<br>• $29^{3/7}$ weeks of gestation<br>• gravida 10 para 7<br>• Pyelonephritis at 13 w.o.g<br>• GDM	• Fever (38,2 degree), dyspnea, myalgias, dysuria, cough<br>• Respiratory status worsen (by admission:95% to 92%), respiratory rate of 23/min, heart rate 109 (oxygen through venturi mask)<br>• Transaminitis and hypertriglyceridaemia because of medication<br>• Tachypneic<br>• D-dimer peaking at 3037 ng/mL	• Status improved rapidly post-caesarean<br>• 2 hours after delivery SpO2 to the low 90th percentile on room air, which improved to 100% on 15 L/min of oxygen<br>• by postoperative day 2, she was weaned to 4 L/min of oxygen via nasal cannula<br>• CT showed extensive bilateral patchy ground glass infiltrates and small consolidations<br>• Uncomplicated C-section	• ICU admission with supplemental oxygen<br>• Birth weight 1705g<br>• Early cord clamping<br>• Apgar scores 9-> 9<br>• Required supplement oxygen<br>• CXR on day 3 showed no evidence of pulmonary disease
**Smati et al 2020**	France	Case Report	1	• 36-year-old pregnant women from the Democratic Republic of Congo<br>• presented severe euglycaemic metabolic acidosis and significant ketonaemia (15.3 mmol/L)<br>• GDM (HbA1c at 6.1% (43.2 mmol/mol) on admission)<br>• BMI before pregnancy: 35,2<br>• gravida 9, para7	• Due to suspected preeclampsia, the patient underwent emergency caesarean section<br>• Viral infection might cause euglycaemic ketoacidosis in pregnant women because of stress	• Plasma insulin, C-peptide, glucagon and free fatty acid levels were assessed 2 days after delivery, showed no indications of insulin deficiency or metabolic failure<br>• ICU admission because of abdominal pain with nausea and vomiting associated with non-febrile dyspnea	• Birth weight 2445g

Author, Year	Location	Study design	Pregnant women, n	Patient characteristics and medical history	COVID-19	Maternal outcomes	Offspring outcomes
**Uzel / Lakhno 2020**	Turkey	Case Report	1	• 35-year-old pregnant women (31 w.o.g.)<br>• GDM (Diagnosed at 24 weeks o.g.)<br>• Tested positive for Covid-19	• Dry cough, fever (38.2), dizziness<br>• Blood pressure 130/80 mm Hg<br>• Rate of respiratory 20 / minute<br>• Obese (BMI 40 kg/m^2)<br>• Lymphopenia, hyperglycemia, trace of proteinuria and ketonuria were detected	• Leukocytosis (19×10^3/ µL) and mild anemia (hemoglobin was 10.1 g/dL) were found in the clinical blood test.<br>• increased variables of serum urea, serum creatinine, serum ALT, serum AST, serum alka- line phosphatase were detected<br>• activated partial thromboplastin time, prothrombin time and international normalized ratio were prolonged. Levels of C-reactive protein (179.7 mg/L) and procalcitonin (0.15 ng/ml) were raised. Hemodynamics was not stable (blood pressure 70/40 mm Hg, pulse rate 130 per minute).<br>• Low saturation (75%)<br>• ICU admission<br>• Maternal death	• fetal distress<br>• Apgar 1 -> 3<br>• 1900g<br>• NICU admission<br>• Early neonatal sepsis<br>• Negative tested for Covid-19
**Tang et al 2020**	China	Case Report	1	• symptomatic, 34-years old, GDM (insulin treatment)	• fever, shortness of breath, dyspnea, negative PCR for SARS-CoV-2; positive IgM and IgG antibodies, in both lungs scattered patchy lesions and small amount of left-sided pleural effusion	• No serious complications at delivery<br>• C-section	• born at 37^{+6}, 4,000g birth weight, Apgar scores 9 -> 10, yellowish skin on second day, bilirubin of 11.3 mg/dL; negative tested; on day 7 IgG positive
**D'Ambrosi et al 2020**	Italy	Retrospective single center study	6	• C1: 32-years-old, Caucasian, Insulin requiring, Gestational Age at Covid-19 diagnosis: 38+1, 6 days hospitalized<br>• C2: 26-years-old, Diet, Magreb, Gestational age at COVID-19 diagnosis: 38+2, 7 days hospitalized<br>• C3: 27-years-old, Magreb, Age at Covid-19 diagnosis: 33+1, 6 days hospitalized<br>• C4:37-yaers-old, Hispanic, Diet, Age at Covid-19 diagnosis: 39+0, 11 days hospitalized<br>• C5: 32-years-old, Magreb, Diet, Age at Covid-19 diagnosis: 29,+1, 49 days hospitalized<br>• C6: 40-years-old, Caucasien, Insulin requiring, 7 days hospitalized, Age at Covid-19 diagnosis: 27+1	• 4/6 had COVID-Symptoms (fever, dyspnea)<br>• Two were asymptomatic	• Two vaginal deliveries, 4 elective C-Sections<br>• 5/6 multiparous<br>• 2/6 Radiological signs<br>• No ICU requiring or mechanical ventilations<br>• One woman required hospitalization for more than 7 days	• C1: birth weight 3670g, Apgar at 5 min: 10<br>• C2: birth weight 3000g, Apgar at 5 min: 10<br>• C3: birth weight 3766g, Apgar at 5 min: 10<br>• C4: birth weight 3766g, Apgar at 5 min: 10<br>• C5: birth weight 2095g, Apgar at 5 min: 10<br>• C6: birth weight 3640g, Apgar at 5 min: 10
**Fontanella et al 2020**	Netherlands	Letter	1	• 38-years-old Caucasian pregnant women, diet controlled GDM,BMI 46, admitted to hospital at 31^{+6} weeks	• COVID symptoms: dry cough, dyspnea, oxygen desaturation (94 on 3 Liter of oxygen), thoracic pain with deep breathing,<br>• CPR, LDH, Glucose levels increased<br>• Needed oxygen support and molecular weight heparin	• Day 2 and 3 betamethasone administered and further conservative management<br>• Thromboprophylaxis started from day 1<br>• No adverse outcome reported, mother left hospital after 5 days because of rapid improvement	• No adverse outcome<br>• Fetal lung maturations prevented by corticosteroids

Author, Year	Location	Study design	Pregnant women, n	Patient characteristics and medical history	COVID-19	Maternal outcomes	Offspring outcomes
					• CT scan found bilateral ground glass opacities suggestive of COVID-19 pneumonia<br>• First nasopharyngeal swab was negative, second positive		
**Gidlöf et al 2020**	Sweden	Letter	1	• 34-yearss-old pregnant women, dichorionic twin pregnancy, BMI 38 kg/m^2 and diagnosed with GDM at 29 weeks, hospitalized at $36^{+2/7}$ weeks, severe preeclampsia	• COVID symptoms: mild headache, hoarseness, increasing malaise at admission<br>• oxygen saturation 96%, respiration rate 11/min., body temperature 38,0 degree, BP 170/100 mm HG heart rate 85 beats7min.,<br>• Creatinine: 89 µmol/L (reference <90 µmol/L) and uric acid 510 µmol/L (reference 155-350 µmol/L).<br>• 7 hours after hospitalization severe headache, photophobia, brisk patellar reflex<br>• On third day, oxygen dropped to 87%, CT showed typical COVID-19 pneumonia signs	• Three hours after delivery positive COVID-19 test result<br>• BP remained stable, the temperature normalized after first postoperative day<br>• On 3rd day, oxygen saturation dropped to 87%, which was managed successfully with oxygen, 1-3 L/min via a nasal cannula<br>• CRP reached 88 mg/L on 4th day but fell to 38 mg/L on the 6th day<br>• Test on breastmilk and maternal vaginal secretion on the 5th day were negative<br>• Emergency C-section, three hours later positive COVID test	• Babies (two girls) were delivered in good condition<br>• Because of GDM, both babies were formula-fed<br>• C1: 2680g, Apgar score 9,10 and 10 at 1,5 and 10 minutes, developed breathing problems at 22 minutes, cyanotic attack while feeding on day two<br>• C2: 2160g, Apgar 9,10 and 10<br>• Both twins had negative nasopharyngeal swab
**Majachani et al 2020**	Grenada	Case Report	1	• 31-year-old Hispanic women with SARS-CoV-2, HIV positive, GDM<br>• C-Section at 34 w.o.g	• COVID symptoms: shortness of breath and myalgia (mild symptoms)<br>• White blood cell count of 10.000 sells/mm3<br>• Chest X-ray showed bibasilar opacities		• Initial examination was normal<br>• Apgar score 9/10 at1 and 5 mins.<br>• NICU admission (for 10 days) for hypoglycemia monitoring and continuous cardiac, respiratory and pulse oximetry monitoring<br>• Nasopharyngeal swab after 24 h positive (no signs of respiratory distress and 95% oxygen)
**Pulinx et al 2020**	Belgium	Case Report	1	• 30-year.old women (gravida 2, para 1)<br>• hospitalized at 22 w.o.g, pregnant with dichorionic diamniotic twins, GDM<br>• Negative tested for toxoplasma IgG and IgM antibodies at 21 w.o.g<br>• Tested positive for SARS-CoV-2 RT-PCR on a nasopharyngeal swab<br>• Preterm birth	• Rhinitis and fever (39,2 degree), oxygen saturation 98%, X-Ray showed no abnormalities<br>• Maternal Blood samples were tested positive for SARS-CoV-2<br>• Aggregates of histiocytes and cytotoxic T lymphocytes in the intervillous space were also present and confirmed (findings supported the diagnosis of chronic intervillositis)	• In the fetal circulation, nuclear debris and an increase in erythroblasts (as can be seen in fetal hypoxia)	• The fetal demise of fetus one was diagnosed and fetus two showed fetal heart rate decelerations<br>• Prepartal intrauterine death of fetus one and prepartal death of fetus two
**Kleinwechter et al 2021**	Germany	Case series	21	• Median age 34 years<br>• 1/3 was obese at the beginning of pregnancy	• Half of the women were asymptomatic (11/21)	• In 2 cases admission to the intensive care unit, and in 4 cases the women	• 17 live births (at the time of the evaluation) of which 1 time twins, median in the 39th week of pregnancy, no early births

Author, Year	Location	Study design	Pregnant women, n	Patient characteristics and medical history	COVID-19	Maternal outcomes	Offspring outcomes
				• GDM diagnosed at 24-28 weeks<br>• SARS-Cov-2 diagnosed at median 38 weeks	• Most common symptoms: cough (6/21), tiredness (6/21), malaise (6/21), nasal congestion (7/21)changes in smell and taste (5/24)<br>• Mostly mild to moderate course in women with GDM	received a symptomatic COVID-19-associated therapy<br>• No invasive ventilation<br>• 56 % sectio (9/16)	
**Rabiei et al 2021**	Iran	Case report	1	• 38 year-old Iranian with triplet<br>• Blood pressure 140/90 mmHg<br>• Elevated liver enzymes<br>• Pregnant by ovulation induction<br>• Hpothyriodism<br>• 16 units insulin daily<br>• HbA1c 5.6%	• Mild fever<br>• No shortness of breath, diarrhea, nausea or vomiting<br>• Oxygen saturation above 96%	• Cesarean section	• First baby: birth weight: 1320g (5-min APGAR was 4)<br>• Second baby:: birth weight 1600g (5-min APGAR was 7)<br>• Third baby: birth weight 1250g (5-min APGAR was 6)<br>• Admission to Neonatal intensive care unit<br>• All three: symptoms of spsis and pulmonary hemorrhage<br>• COVID-19 test was negative for all three
