# Supplementary material for: Telemedical Approaches to Managing Gestational Diabetes Mellitus During COVID-19: Systematic Review
Source: JMIR Pediatr Parent. 2021 Aug 5;4(3):e28630. doi: 10.2196/28630 (PMC8345174; doi:10.2196/28630)
Supplement: Multimedia Appendix 4 [file pediatrics_v4i3e28630_app4.pdf]

Quality assessments.

Question/ Trial	A Seletion Bias (Q1)	A Selection Bias (Q2)	A SCORE	B Study Design	B SCORE	C Confoun-ders (Q1)	C Confoun-ders (Q2)	C SCORE	D Blinding (Q1)	D Blinding (Q2)	D SCORE	E Data collection (Q1)	E Data Collection (Q2)	E SCORE	F Withdrawals and Drop-outs (Q1)	F Withdrawals and Drop-outs (Q2)	F SCORE	GLOBAL RATING
Perez-Ferre et al 2010	1	1	**	1	***	2	/	***	3	3	**	1	1	***	1	1	***	**Strong**
Dalfra et al 2009	1	1	**	1	***	2	/	***	1	1	*	1	1	***	1	1	***	**Strong**
Guo et al 2019	1	1	**	1	***	2	/	***	1	1	*	1	1	***	1	1	***	**Strong**
Homko et al 2007	1	1	**	1	***	2	/	***	1	3	**	1	1	***	1	1	***	**Strong**
Given et al 2015	1	2	**	1	***	2	/	***	1	1	*	1	1	***	1	1	***	**Moderate**
Homko et al 2012	1	5	*	1	***	2	/	***	3	3	**	1	1	***	1	1	***	**Moderate**
Mackillop et al 2018	1	1	**	1	***	2	/	***	3	3	**	1	1	***	1	1	***	**Moderate**
Yang et al 2018	1	5	*	1	***	2	/	***	3	3	**	1	1	***	1	1	***	**Moderate**
Kim et al 2019	1	5	*	2	***	2	/	***	1	1	*	1	1	***	1	1	***	**Weak**

* = week, ** = moderate, *** = strong, 1-5 = response options according to EPHPP, / = not applicable
