# Supplementary material for: Telemedical Approaches to Managing Gestational Diabetes Mellitus During COVID-19: Systematic Review
Source: JMIR Pediatr Parent. 2021 Aug 5;4(3):e28630. doi: 10.2196/28630 (PMC8345174; doi:10.2196/28630)
Supplement: Multimedia Appendix 6 [file pediatrics_v4i3e28630_app6.pdf]

Forest plot using HbA$_{1c}$ values (%) at the end of the study

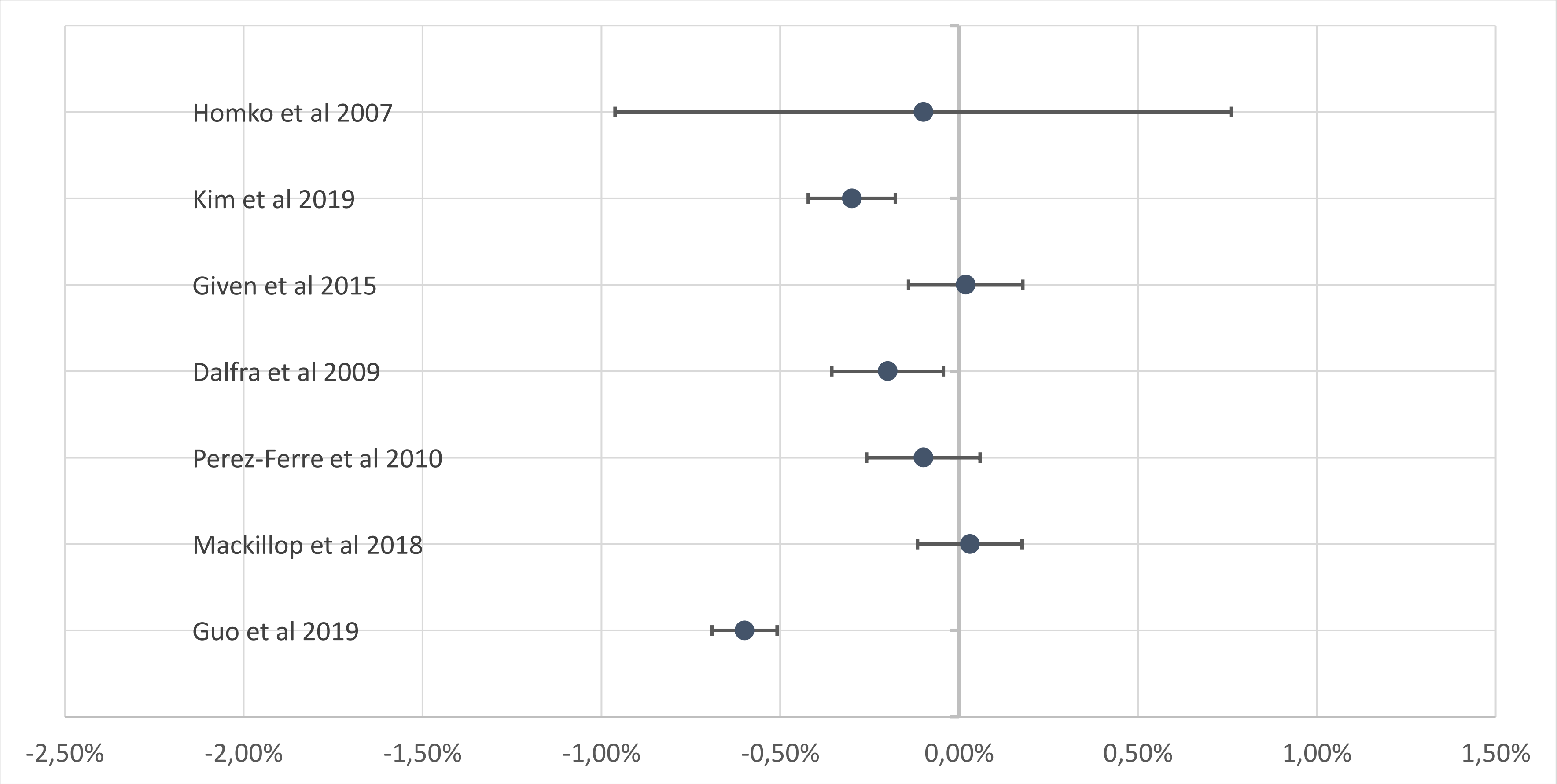

Calculations and data for meta-analysis

		n	Mean	Std		Effect size	Pooled Std**	t	CI***	plot line	plot labels
Homko et al 2007	IG	34	6,10%	0,80%		-0,10%	0,43%	2,000	0,86%	7	-2,20%
Homko et al 2007	CG	29	6,20%	2,20%							
Kim et al 2019	IG	22	5,00%	0,20%		-0,30%	0,06%	2,018	0,12%	6	-2,20%
Kim et al 2019	CG	22	5,30%	0,20%							
Given et al 2015	IG	24	5,26%	0,30%	*	0,02%	0,08%	2,011	0,16%	5	-2,20%
Given et al 2015	CG	26	5,25%	0,26%	*						
Dalfra et al 2009	IG	88	5,10%	0,60%		-0,20%	0,08%	1,972	0,16%	4	-2,20%
Dalfra et al 2009	CG	115	5,30%	0,50%							
Perez-Ferre et al 2010	IG	50	5,30%	0,40%		-0,10%	0,08%	1,984	0,16%	3	-2,20%
Perez-Ferre et al 2010	CG	50	5,40%	0,40%							
Mackillop et al 2018	IG	42	5,42%	0,34%		0,03%	0,07%	1,988	0,15%	2	-2,20%
Mackillop et al 2018	CG	46	5,39%	0,35%							
Guo et al 2019	IG	64	4,70%	0,20%		-0,60%	0,05%	1,980	0,09%	1	-2,20%
Guo et al 2019	CG	60	5,30%	0,30%							
